# Considerable use of Furazolidone in Iran

**DOI:** 10.4103/1319-3767.70631

**Published:** 2010-10

**Authors:** Amin Talebi Bezmin Abadi, Tarang Taghvaei, Dino Vaira

**Affiliations:** Department of Bacteriology, School of Medical Sciences, Tarbiat Modares University, Tehran; 1 Department of Internal Medicine, Faculty of Medicine, Mazandaran University of Medical Sciences, Sari, Iran, E-mail: ttaghvaeei@mazums.ac.ir; 2 Department of Internal Medicine and Gastroenterology, S. Orsola Hospital, Massarenti Bologna, Italy

Sir,

Recently, we read a paper by Hasan *et al*.,[1] suggesting that application of furazolidone in combination with amoxicillin and omeprazole and bismuth subcitrate for two weeks can be a good choice for *Helicobacter pylori* eradication for dyspeptic patients from Yazd, Iran. However, based on our last publication[2], there are some contradictory findings related to their publication in *Saudi Journal of Gastroenterology*. Prescribing furazolidone, a new antibiotic for eradication of *Helicobacter pylori* from the stomach of infected individuals,[3] is still a matter of controversy. Two major topics concerning the widespread use of furazolidone in Iran remain unclear. We have isolated a high rate of resistant strains of *H. pylori*, as reported in our publication in 2009,[2] which is close to 25% resistance rate for furazolidone. This is high for a new drug in clinical practice. It was expected that furazolidone would not have a high rate of resistance because it has been newly introduced in our country and *H. pylori* have not been extensively exposed to this antibiotic.

We believe that the Iranian medical community cannot rely on this drug for achieving an effective regimen against *H. pylori* strains. New data indicating a rising antimicrobial resistance among *H. pylori* isolates has been seen. This phenomenon has forced us to find new interventions for optimum treatment. Knowing the status of antibiotics resistance to *H. pylori* can help physicians to determine best treatment of choice against this rogue bacteria.[3] Furthermore, comprehensive follow-up procedures for primary treated patients,[3] especially with drugs such as furazolidone -as a newly proposed treatment- will be helpful for effective eradication of *H. pylori*.

Second, Hasan *et al*,[1] used negative rapid urea test (RUT) as confirmation of eradicated infection, a method which has not been recommended by European as well as American guidelines.[4,5] Results of our new study [data not published] revealed that rising resistance for furazolidone in our *H. pylori* isolates showed that we are in need of emergency efforts for solving this problem in our country. Until further trials are performed including follow-up with a high sample size for each region and identification of the exact pattern of resistance, we strongly recommend avoiding the use of furazolidone in treatment regimens of *H. pylori*.
